# Collection of cerebrospinal fluid in 50 adult healthy donkeys (*Equus asinus*): clinical complications, and cytological and biochemical constituents

**DOI:** 10.1186/s12917-021-03007-4

**Published:** 2021-09-09

**Authors:** Mohammed A. H. Abdelhakiem, Hussein Awad Hussein

**Affiliations:** 1 Department of Animal Surgery, Anesthesiology and Radiology, Faculty of Veterinary Medicine, Assiut University, Assiut, 71526 Egypt; 2 Internal Veterinary Medicine, Department of Animal Medicine, Faculty of Veterinary Medicine, Assiut University, Assiut, 71526 Egypt

**Keywords:** Biochemistry, Cerebrospinal fluid, Clinical complications, Cytology, Donkey

## Abstract

**Background:**

Diseases of the central nervous system are a well-recognized cause of morbidity and mortality in equine. Collection and analysis of cerebrospinal fluid (CSF) give information about the type and stage of degenerative and inflammatory diseases in central nervous system (CNS). The present research aimed to assess the clinical complications of CSF collections and to establish range values of cytological and biochemical parameters of CSF in adult healthy donkeys (*Equus asinus*). The CSF samples were collected from fifty healthy donkeys at the lumbosacral (LS) and atlanto-occipital (AO) sites.

**Results:**

Hypothermia, tachycardia, ataxia and recumbency may develop post-puncture. Erythrocytes were noticed in 35 of 50 CSF samples. Total nucleated cell counts ranged from 0 to 6 cells/μL, and lymphocytes predominated the cells (61%). The concentration of glucose (1.2 to 5.3 mmol/L) was lower than that of serum (*P* < 0.05). The CSF sodium concentration (123 to 160 mmol/L) was approximately like that of serum, but potassium (1.5–3 mmol/L) was lower than that of serum (*P* < 0.01). Urea concentrations (1.1–2.9 mmol/L) were markedly lower than serum (*P* < 0.001). Concentrations of CSF total proteins, and albumin ranged from 0.1 to 0.6 g/dL, and from 0.002 to 0.013 g/dL, respectively. The albumin quotient ranged from 0.06 to 0.56.

**Conclusions:**

Transient hypothermia, tachycardia, ataxia and recumbency may develop as clinical complications of CSF puncture procedures. The collection site has no impact on the constituents in CSF. Furthermore, this study presented the range values for normal cytological and biochemical constituents of CSF in donkeys (*Equus asinus*) that can provide a basis in comparison when evaluating CSF from donkeys with neurologic diseases.

## Background

Diseases of the central nervous system are a well-recognized cause of morbidity and mortality in equine [1]. Many diseases can affect equine central nervous system (CNS), including equine protozoal myeloencephalopathy, equine degenerative myeloencephalopathy, and equine herpesvirus-1 myeloencephalopathy [2]. Analysis of CSF gives information about the type and stage of inflammatory or degenerative processes occurring in CNS [3]. CSF is the fluid that flows in and around the hollow spaces of the brain and spinal cord, and between the meninges [4]. CSF originates from the choroid plexus and ependymal lining of the ventricles [5], and it flows from the ventricular system up over the cerebral hemispheres and through the subarachnoid space surrounding the spinal cord [6]. The pulse waves of the blood in the choroid plexuses push the CSF in a caudal direction [7]. CSF has many functions, including physical support of the brain and spinal cord, excretion action, intracerebral transport, and maintenance of chemical environment of the CNS [7], including proper ionic and acid-base balance [8]. Some body barriers as the blood - brain barrier, the blood-CSF barrier, and the CSF-brain barrier control the composition, production and absorption of the CSF [9].

In equine, CSF can be collected via the atlanto-occipital (AO) or lumbosacral (LS) spaces [2]. Many factors affect the practitioner’s choice as LS puncture is more likely to give information about diseases posterior to the foramen magnum [10], because CSF has caudal flow [11]. Analysis of cerebrospinal fluid (CSF) is a useful method for the diagnosis of equine with suspected CNS disease [2]. In addition, CSF analysis has reasonable sensitivity but low specificity, as well as the CSF abnormalities are usually dependent on the CSF collection site with respect to the lesion location [12].

At present, according to the authors’ knowledge, there are no literatures describing the clinical complications of CSF puncture procedures in donkeys. Therefore, the objectives of the current research were as follows: [1] describe the clinical observations and possible complications that may develop after CSF collection in clinically healthy adult donkeys (*Equus asinus*); [2] determine the cytological and biochemical constituents of CSF analysis.

## Materials and methods

### Animals and study design

The present research was ethically approved by the Animal Care and Welfare Committee of Faculty of Veterinary Medicine, Assiut University, Assiut, Egypt. All national and institutional guidelines for the care and use of animals were followed during study procedures. All animals were housed and cared according to the Egyptian animal welfare act (No. 53, 1966). Moreover, informed consent was granted by the owners of the donkeys. The current study was carried out on 50 adult clinically healthy donkeys (*Equus asinus*), of both sexes (28 males, and 22 non-lactating and non-pregnant females). The average age was 8 ± 1.5 years, and weight was 110 ± 2.8 kg (mean ± standard error). All animals were clinically healthy and showed no signs of CNS or other neurological diseases during physical examination. The body score of animals ranged from 2 to 3 [13]. The clinical examination including rectal temperature, heart rate, and respiratory rate were conducted for all animals [14], as well as the mental status, behavior, posture, gait, involuntary movement, and lameness were recorded pre- and post-collection of CSFs. Animals showed any clinical and/or neurological abnormalities were removed from the study. All animals were kept under clinical observation for 72 h post-collection for recording any abnormalities that may develop post-punctures. All donkeys were housed in a free stable yard with feed and water ad libitum.

### Anesthesia and CSF collection

**Fig. 1:**
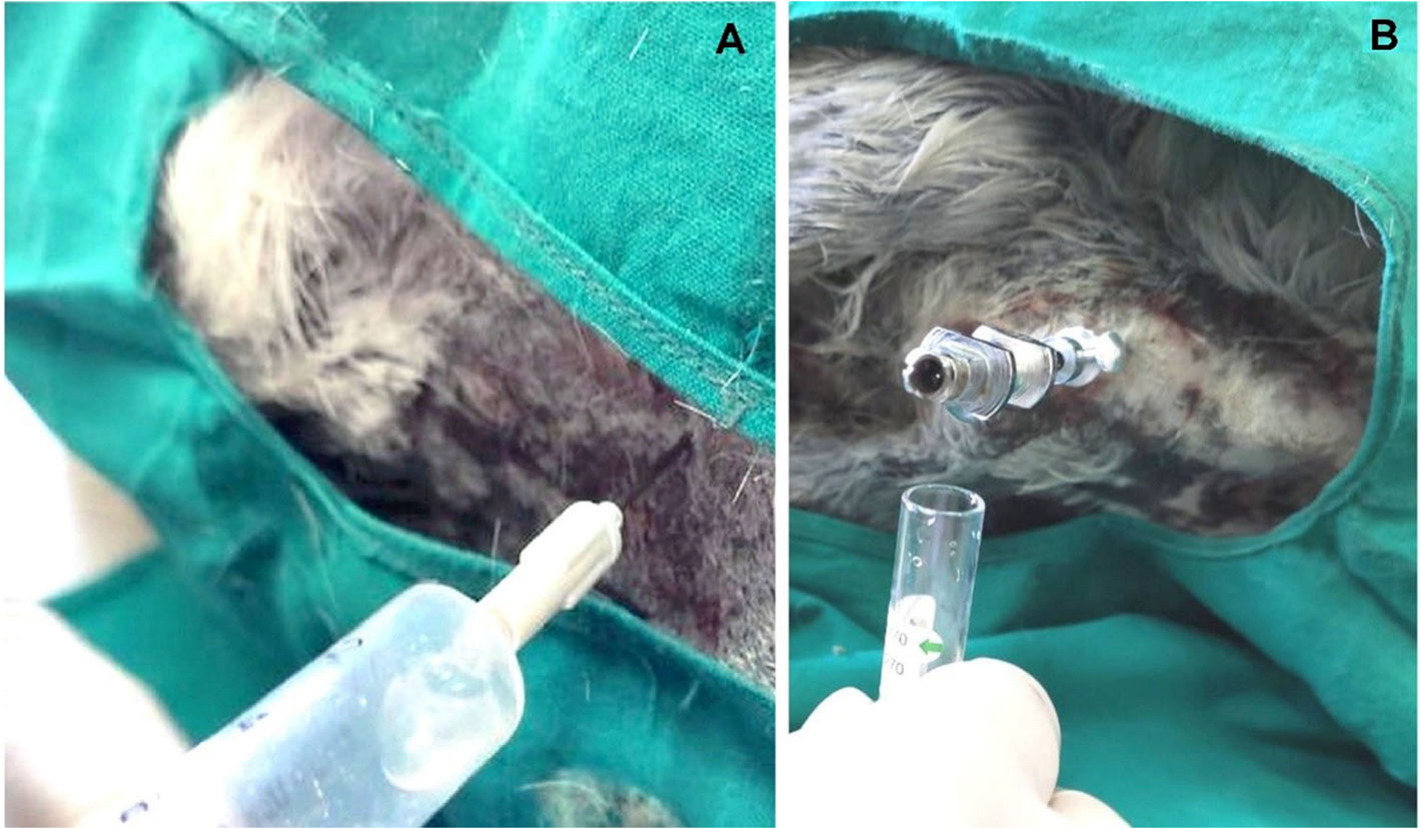
Collection of CSF via the LS puncture in a recumbent donkey (**A**). Atlanto-occipital puncture and CSF collection, where the neck is flexed and the needle is inserted perpendicular to the long axis (**B**)

For induction of anesthesia, intravenous (IV) 0.03 mg/kg acepromazine (Calmivet 5 mg/ml, Vetoquinol, Grovet Health Company, Utrecht, Netherlands), and 15 min thereafter, each donkey was injected IV with 1 mg/kg xylazine 2% (Xyla-Ject, ADWIA Co., Egypt). The anesthesia was maintained by intramuscular injection of 2 mg/kg ketamine (Ketamine Rotexmedica 50 mg/ml, Arzeneimittelwerk GmbH Rotexmedica, Germany). From each donkey, two CSF samples were collected, the first was obtained from lumbosacral site, and the second sample was collected from atlanto-occipital place [15, 16]. Both collections were taken approximately on the same time. The puncture sites were aseptically prepared as usual through clipping and shaving of the hair, then scrubbing of the field using ethyl alcohol 70 and 10% povidone-iodine solution. For lumbosacral puncture, the site was detected at the midline in the space between the cranial ends of sacral tuberosities, about 2–3 cm caudal to the spinous process of the last lumbar vertebra. Samples of CSF were collected in a right recumbent position. The spinal needle was gently inserted till puncturing the arachnoid space, where the animal showed arching of back, contraction of the abdominal muscles, and raising of the tail, then the fluid was noticed in the needle hub (Fig. 1A), and 5 ml was collected in a clean tube. In both techniques, 18-gauge, 10 cm sterile spinal needles were used. For collection of CSFs from atlanto-occipital site, all animals were laterally recumbent on the right side, then the head of animals was flexed. The spinal needle was inserted gently into the subarachnoid space until the CSF appeared at the needle hub. In a clean tube, 5 ml of the fluid were collected (Fig. 1B). For management of pain, each donkey was injected IV by 0.6 mg/kg meloxicam every day for three successive days’ post-collection.

### Blood sampling and laboratory analyses

A one-time blood sample was collected from each donkey by venipuncture from the jugular vein immediately following CSF collection. After that, the blood samples were centrifuged for 15 min according to centrifuge equation: RCF = (RPM/1000)^2^ × r × 1.118, where RCF = relative centrifugal force, RPM = number of rotations per minute, r = centrifuge radius, thereafter the sera were harvested and analyzed for biochemical indices.

For cytological analysis, the samples of CSF were examined immediately after collection. Total nucleated cell count was determined using a hemocytometer as reported elsewhere [17]. For differential cell count, the CSF sample was centrifuged, and then a film was prepared from the sediment and stained with Giemsa’s stain [18]. The supernatants of CSF samples were used for measurement of biochemical parameters. The concentrations of glucose, sodium, potassium, chloride, calcium, inorganic phosphorus, magnesium, urea, total proteins, and albumin in serum and CSF samples were determined using commercial test kits and a spectrophotometer (Spectro UV-Vis, USA) according to the instructions of manufacturers. Albumin quotient (AQ) was calculated using the following formula [19]:

$$ \mathrm{AQ}=\frac{\mathrm{CSF}\ \mathrm{albumin}\ \left(\mathrm{g}/\mathrm{dL}\right)}{\mathrm{Serum}\ \mathrm{albumin}\ \left(\mathrm{g}/\mathrm{dL}\right)}\times 100 $$

### Statistical analysis

Data are presented as means ± standard error (SE) and the analysis was carried out using SPSS software (IBM SPSS analytical program for Windows Version 21; SPSS GmbH, Munich, Germany). The normal distribution of all data was tested using Kolmogorov-Smirnov test. To compare the effect of puncture site (AO vs. LS) on each parameter, paired sample *t*-test was used. To compare between AO and LS methods, linear regressions were carried out, and R square and regression coefficients were estimated, as well as Bland-Altman analysis was conducted. For all statistical procedures, the degree of significance was set at *P* < 0.05.

## Results

### Post-puncture clinical findings and complications

**Fig. 2:**
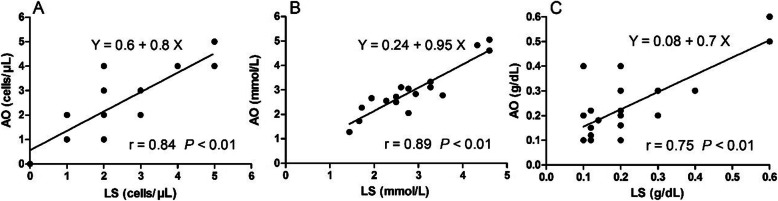
Scatter plots for the regression analysis of white blood cells (**A**), glucose (**B**), and total proteins (**C**) in AO and LS puncture sites

Table 1 summarizes the main clinical findings in donkeys after collection of CSFs. Physical examination revealed a significant decrease of body temperature post-puncture (*P* < 0.05). However, the rectal temperature returned to the normal physiological values 2 h post-collection of CSF samples. In contrast, heart rates showed significant increases post-puncture (P < 0.05), while the respiratory rates showed insignificant changes (*P* > 0.05). Five donkeys suffered from hind limb ataxia and recumbency and were unable to stand by themselves after AO puncture. However, they walked sound without difficulties 2 h thereafter. The feed and water intakes were normal after collection, however, three donkeys showed local pain, discomfort, shaking of the tail (Fig. 2), depression and inappetence after LS puncture, however, these signs disappeared 1 h later. In addition, haemorrhage, spinal hematoma and CNS herniation were not noticed post-puncture.

**Table 1:** Clinical findings ^a^ and complications in donkeys post CSF punctures

Variables	Pre-sampling of CSF	Post-sampling of CSF
Temperature (°C)	37.3 ± 0.6	36.6 ± 0.3^b^
Range (min – max)	(37–37.7)	(36.4–37.3)
Numbers (n)	50	50
Heart rate (beats/min)	34 ± 0.8	45 ± 1.3^b^
Range (min – max)	(26–42)	(29–60)
Numbers (n)	50	50
Respiratory rate (cycle/min)	17.5 ± 0.2	18.2 ± 0.6
Range (min – max)	(13–22)	(14–24)
Numbers (n)	50	50
Main clinical observations (number of donkeys)	The appetite of all animals was normal with normal gait and posture. (50)	1. Five donkeys suffered from hind limb ataxia and recumbency and were unable to stand by themselves after AO puncture. However, they walked sound without difficulties 2 h thereafter. 2. Three cases showed local discomfort, inappetence and depression after LS puncture, and then 1 h later they ate normally.

^a^ Values are mean ± standard error of the mean; ^b^ significance (*P* < .05)

### Laboratory findings

**Fig. 3:**
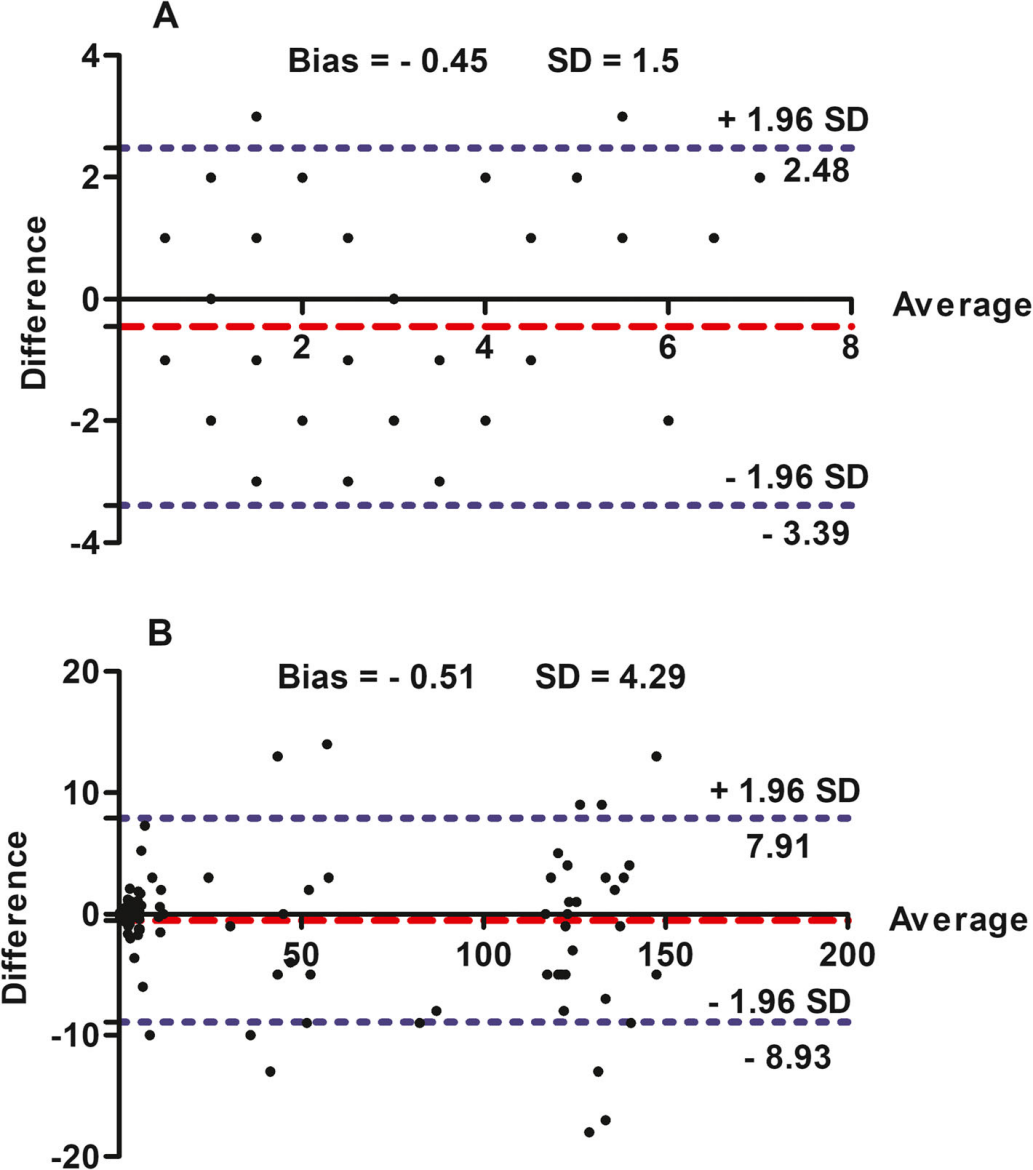
Bland–Altman difference plots for cytological (**A**) and biochemical (**B**) constituents in CSF samples retrieved from AO and LS sites. Y-axis: The constituent value measured in AO site minus the value of the same constituent measured in LS site (Difference). X-axis: Average of the constituent values obtained with the two puncture sites. The mean of the differences or bias (red dashed lines) and the 95% limits of agreement (mean ± 1.96 SD) are included in the graph (blue dotted lines)

In both techniques, most of CSF samples were clear and colorless, and did not clot after collection. Of 50 CSF samples collected using AO puncture, 40 were clear, 7 were slightly turbid and 3 were highly turbid. Of 50 CSF samples collected using LS puncture, 40 were clear, 4 were turbid and red-tinged and 6 highly turbid. In both techniques, all cytological and biochemical analyses were conducted on the clear CSF samples (40 CSF samples). Table 2 shows the results of cytological analysis of CSF samples. The statistical analysis of cellular elements of CSF revealed no significant difference between AO and LS samples (*P* > 0.05). The results of biochemical findings of serum and CSF in clinically healthy donkeys were presented in Table 3. In comparison with serum values, the CSF level of glucose was decreased (*P* < 0.05), while sodium showed no significant changes (P > 0.05). In comparison with their serum values, the CSF concentrations of potassium was lowered (*P* < 0.01), while chloride was higher (P < 0.01). The CSF concentrations of calcium, phosphorous, magnesium and blood urea were lower than their corresponding values serum (P < 0.01). The puncture site had no significant influences on the studied biochemical parameters. Table 4 summarizes the protein profiles in CSF and serum samples. The mean values of AQ in CSF collected from AO and LS sites were 0.21 and 0.23, respectively (*P* > 0.05). The correlation coefficients (*r*) for cytological and biochemical variables in AO CSF and their corresponding parameters in LS CSF ranged from moderate to strong positive relationships (Table 5), indicating the association of the analyzed parameters in both taps. Figure 2 illustrates the regression analyses of white blood cells, glucose, and total proteins. In addition, the Bland–Altman plots indicated proportional bias between AO and LS taps for cytological (bias = − 0.45, SD = 1.5; Fig. 3A) and biochemical (bias = − 0.51, SD = 4.29; Fig. 3B) constituents, and 95% limits of agreements were also included.

**Table 2:** Cytological constituents of cerebrospinal fluid in donkeys (*Equus asinus*) (*n* = 40)

Variables	Cerebrospinal fluid						*P*-value
	Atlanto-occipital			Lumbosacral			
	mean ± SE	Range	95% CI^*^	mean ± SE	Range	95% CI^*^	
Red blood cells (cells/μL)	4.5 ± 0.5	0–9	3.4–5.7	4.3 ± 0.5	0.0–8.0	3.2–5.5	0.787
White blood cells (cells/μL)	2.1 ± 0.3	0–6	1.5–2.7	2 ± 0.3	0–5	1.3–2.7	0.836
Lymphocytes (%)	61 ± 10	49–69	55–70	59.6 ± 10	42–73	51–67	0.719
Monocytes (macrophages) (%)	35 ± 6	24–48	28–42	38.4 ± 7	26–57	32–48	0.704
Neutrophils (%)	4 ± 2	0–8	3–5	2 ± 1.8	0–7	1–3	0.832

^*^CI, Confidence interval

**Table 3:** Cerebrospinal fluid and serum reference biochemical findings in donkeys; (*Equus asinus*) (*n* = 40)

Variables	Cerebrospinal fluid						P-value	Serum		
	Atlanto-occipital			Lumbosacral						
	mean ± SE	Range	95% CI^*^	mean ± SE	Range	95% CI^*^		mean ± SE	Range	95% CI^*^
Glucose (mmol/L)	2.8 ± 0.3	1.2–5.3	2.3–3.4	2.7 ± 0.2	1.4–4.6	2.2–3.2	0.364	3.9 ± 0.3	1.9–5.1	3.3–4.6
Sodium (mEq/L)	136 ± 2	123–150	132–140	138 ± 4.4	125–160	128–147	0.740	140 ± 2	120–150	136–144
Potassium (mEq/L)	2.2 ± 0.1	1.5–3	1.9–2.5	2 ± 0.1	1.5–3	1.8–2.2	0.199	4.1 ± 0.2	3–5	3.8–4.4
Chloride (mEq/L)	122 ± 0.8	117–126	120–124	121 ± 1	115–126	119–123	0.421	105 ± 0.8	100–110	103–106
Calcium (mmol/L)	1.3 ± 0.03	1.3–1.6	1.2–1.4	1.4 ± 0.04	1.1–1.7	1.3–1.4	0.746	2.9 ± 0.05	2.6–3.2	2.7–3.0
Phosphorus (mmol/L)	0.8 ± 0.04	0.6–1.1	0.7–0.9	0.8 ± 0.03	0.5–1.0	0.7–0.9	0.870	1.7 ± 0.05	1.5–2.0	1.6–1.8
Magnesium (mmol/L)	0.7 ± 0.03	0.5–0.9	0.6–0.7	0.6 ± 0.02	0.5–0.8	0.6–0.7	0.675	1.2 ± 0.05	0.8–1.2	1.1–1.4
Urea (mmol/L)	2.0 ± 0.1	1.3–2.9	1.6–2.1	1.9 ± 0.1	1.1–2.9	1.5–2.1	0.962	16 ± 0.4	14–19	16–17
Specific gravity	1004 ± 0.3	1003–1007	1004–1005	1005 ± 0.5	1003–1010	1004–1006	0.787	NA	NA	NA

^*^CI, Confidence interval

**Table 4:** Cerebrospinal fluid (CSF) and serum protein profiles in donkeys (*Equus asinus*) (n = 40)

	Cerebrospinal fluid						***P***-value			
	Atlanto-occipital			Lumbosacral				Serum		
Variables	mean ± SE	Range	95% CI^*^	mean ± SE	Range	95% CI^*^		mean ± SE	Range	95% CI^*^
Total proteins (g/dL)	0.23 ± 0.04	0.1–0.6	0.15–0.3	0.19 ± 0.03	0.1–0.6	0.1–0.26	0.213	7.8 ± 0.3	6–9	7.2–8.3
Albumin (g/dL)	0.008 ± 0.001	0.003–0.013	0.005–0.01	0.007 ± 0.001	0.002–0.012	0.005–0.01	0.841	3.5 ± 0.12	2.4–4	3.2–3.7
Albumin quotient	0.23 ± 0.04	0.1–0.56	0.15–0.30	0.21 ± 0.03	0.06–0.36	0.15–0.27	0.709	NA		

^*^CI, Confidence interval

**Table 5:** The regression analysis between the cytological and biochemical constituents in AO and LS CSF samples

Parameters	Regression analysis results		
	R^2^	P-value of regression	Coefficients of regression
AO Red blood cells – LS Red blood cells	0.44	0.004	0.35
AO Lymphocytes – LS Lymphocytes	0.52	0.003	0.44
AO Monocytes – LS Monocytes	0.34	0.022	0.30
AO Neutrophils – LS Neutrophils	0.67	0.001	0.52
AO Sodium – LS Sodium	0.61	0.002	0.43
AO Potassium – LS Potassium	0.42	0.004	0.37
AO Chloride – LS Chloride	0.64	0.003	0.62
AO Calcium – LS Calcium	0.58	0.003	0.48
AO Phosphorous – LS Phosphorous	0.46	0.005	0.41
AO Magnesium – LS Magnesium	0.55	0.003	0.39
AO Urea – LS Urea	0.42	0.004	0.36
AO Specific gravity – LS Specific gravity	0.31	0.025	0.28
AO Albumin – LS Albumin	0.59	0.003	0.52
AO Albumin quotient – LS Albumin quotient	0.56	0.003	0.48

## Discussion

The collection and analysis of CSF can provide valuable information about the central nervous system. Evaluation of CSF is an important tool, and together with the history, clinical examination, neurologic examination, and other ancillary test procedures may help in the diagnosis and prognosis of neurologic disease in equine [19].

In the present work, CSF samples, in both techniques, were collected from anesthetized animals with analgesic drug as reported in methodology. It had been recommended induction of general anesthesia for AO puncture [20] and sedation for LS tap [21]. In contrast, anesthesia has very complex direct and indirect influences on cerebral blood flow and cerebral function [22].

In the current study, physical examination revealed lowered body temperature after collection of CSF, indicating a clinical complication and a physiological response in stressed donkeys. In a previous study [23], the authors reported that fear may evoke cutaneous vasoconstriction with subsequent minimal reduction of body temperature. Furthermore, the increased heart rate could be explained because of fear, excitement and pain of puncture. It had been reported that painful stress and fear are usually accompanied with release of catecholamines with resultant tachycardia [24]. However, the variation of these two clinical variables was transient in the present study as the values of temperature and heart rates returned to the physiological limit [25] within 2 h post-sampling. In this research, few animals showed ataxia and recumbency post-puncture of LS. This finding could be attributed to the pain sensation during needle insertion causing the animal struggle with resultant incoordination. However, the animals walked sound later, indicating no permanent injuries were made in the spinal cord during puncture. This postulation was supported in a previous study [10], iatrogenic trauma may develop as a complication during animal puncture for CSF collection. In the current work, no serious complications were observed post-puncture. In contrast, serious complications including post-puncture infection, hematoma, subdural hemorrhage, and herniation of cerebellum were listed elsewhere [2].

Analysis of CSF is a general index of CNS health and often provides useful information about the type of neurologic lesion that is present [7]. In the current study, CSF samples were clear and colorless. As mentioned elsewhere [9], CSF is formed principally by the choroid plexuses, where hydrostatic pressure of the choroidal capillaries initiates the transfer of water and ions to the interstitial fluid and then to the ventricles through ion pump. In this study, some CSF samples were turbid or red-tinged, indicating blood and/or other tissues contamination during collection procedure. This finding is consistent with those in horses [10] and goats [26].

In both puncture techniques, values of RBCs ranged from 0 to 8 cells/μL, indicating minor blood contamination. Furthermore, blood contamination in this study was not felt to be significant based on clarity, color and small number of RBCs, as well as the all turbid and red-tinged CSF samples were excluded from the analytical procedures to avoid misinterpretation of obtained data. In contrast, no RBCs were observed in CSF samples of another breed of donkey (miniature) [27]. Such difference could be attributed to species and/or collection technique variations. However, it had been listed the counts of RBCs in AO and LS CSF samples of normal horses as 51 and 37 cells/μL, respectively [8].

In the current study, results of cytology, including total nucleated cells and differential cell count of both punctures, are consistent with previous findings in horses [28, 29]. CSF in normal horses contains fewer than 10 white blood cells per microliter [8]. However, many variations may occur in WBCs counts in CSF of equine [29]. In the present research, no significant difference was observed between WBCs counts in AO and LS CSF samples. This finding was supported elsewhere [29], no variations in white blood cell counts in CSF samples taken from AO and LS sites. In contrast, LS site may show a slightly higher WBCs counts than AO but less than 10 cells/μL [19]. In the current work, lymphocytes were the predominant cells, then monocytes and neutrophils with low percentages. This finding was similar to that reported for horses [2, 8].

Biochemically, the CSF glucose concentrations for the donkeys in this study were slightly higher than those previously reported in miniature donkeys [27]. These differences may be due to the association between the last feeding and CSF collection times [7]. However, the concentration of CSF glucose in the current research was approximately 70% of the serum value and similar to horses [28], and dogs [30]. The concentration of sodium in CSF was nearly similar to the value in serum. It had mentioned previously [31], CSF concentration of sodium is considered diagnostic for salt poisoning when its level is higher than 160 meq/L. However, potassium level in CSF was lower than that in serum and was less than a value published in miniature donkeys [27]. The CSF concentration of chloride was higher than that of serum. In donkeys, CSF has a different ionic composition than does serum, containing less potassium, calcium, phosphorus and magnesium and more chloride. In the current study, the serum concentration of urea was higher than that of CSF. This finding was similar to that reported in horses [28] and camels [32]. Furthermore, CSF concentrations of urea were lower than the values reported for goats [26]. As reported elsewhere [33], CSF urea concentration is lower than serum and its values are dependent upon the serum concentration.

In the present study, the concentration of CSF total proteins was nearly in agreement with a previous report in horses [28], and in disagreement with miniature donkeys [27]. Values of total proteins may vary with the technique used for its measurement [34]. Total proteins’ concentration is higher in LS CSF compared with AO CSF [19]. Increased concentration of CSF total protein was recognized as an indicator of neurological disease and/or CNS infection [7]. In the current study, no significant difference was seen between CSF total proteins of AO and LS sites. Furthermore, a strong correlation coefficient of 0.75 was obtained, relating total proteins concentrations in AO and LS CSF samples, indicating no variation between both puncture sites. In contrast, a higher LS CSF total protein concentration compared with AO CSF total proteins had been described in horses [35]. Values of albumin and albumin quotient are generally inconsistent with those reported for the horses [19]. Furthermore, a comparison of collection sites revealed no significant differences. It had mentioned that increased CSF albumin indicated damage to the blood-brain/CSF barriers, intrathecal hemorrhage, or a traumatic CSF tap [1, 7].

In general, some cytological and biochemical constituents of CSF in this study are varied from those reported in miniature donkeys [27] and horses [19]. Such differences could be attributed to species and/or CSF collection technique variations.

Collection of CSF from both sites at the same time and comparing the findings may be helpful in cases in which neuroanatomic localization of the lesion is difficult [36]. In the present study, there is no great difference between the cytological and biochemical composition of AO and LS CSF samples, as well as the parameters of both punctures’ sites were positively associated together with acceptable regression coefficients. This finding is indicating no substantial variation between the constituents of AO and LS samples. Consequently, using either AO or LS sampling sites is feasible for evaluation of CSF in healthy donkeys. However, in diseased animals, CSF should be collected as near as possible to the suspected lesion [1]. In addition, cisternal puncture is indicated when the disease suspicions involving the brain [37], and LS tap is recommended when the suspected diseases including spinal cord [2].

## Conclusions

Minor physical changes in the form of transient hypothermia and tachycardia, as well as ataxia and recumbency may develop as clinical complications of puncture procedures for CSF collection with rapid subsequent recovery. The puncture site had no effect on the cytological and biochemical constituents of CSF samples. The current study presented the normal values for cytological and biochemical constituents of CSF in donkeys (*Equus asinus*) that can provide a basis in comparison when evaluating CSF from donkeys with neurologic diseases.

## Data Availability

The datasets generated and/or analyzed during the current study are available from the corresponding author on reasonable request.
